# Will Innovation of Pharmaceutical Manufacturing Improve Perceived Health?

**DOI:** 10.3389/fpubh.2021.647357

**Published:** 2021-04-29

**Authors:** Zejun Li, Xue Li

**Affiliations:** ^1^School of Computer and Information Science, Hunan Institute of Technology, Hengyang, China; ^2^School of Economics and Statistics, Guangzhou University, Guangzhou, China

**Keywords:** pharmaceutical manufacturing innovation, perceived health, lag effect, heterogeneity, moderating effect

## Abstract

By taking 22 OECD countries from 2010 to 2017 as sample, we study the effect of pharmaceutical manufacturing innovation on perceived health by using the panel Tobit model from the entire sample and sub-samples, respectively, as well as analyze their transmission channels by adding moderating effect. Based on the above, we get the following results: first, the pharmaceutical manufacturing innovation 4 years ago has a positive influence on perceived health, which means the improvement of perceived health is closely related to pharmaceutical manufacturing innovation 4 years ago. Second, pharmaceutical manufacturing innovation has a heterogeneous impact on perceived health, which, including the size and direction of the impact effect, is mainly reflected in different pharmaceutical manufacturing innovation levels, population aging degrees, and education levels. Third, income level can positively regulate the impact of pharmaceutical manufacturing innovation on perceived health.

## Introduction

The level of innovation in the pharmaceutical manufacturing has an important impact on people's health. On the one hand, the important influence of pharmaceutical manufacturing originates from the feature of the pharmaceutical industry itself. The pharmaceutical industry, which is driven by technical innovation, is a typical R&D industry. Generally, pharmaceutical manufacturing is considered as the largest R&D import sector among all the industries (1). Innovation is one of the elements of development for an industry (2). Only by improving the efficiency of technological innovation can the pharmaceutical manufacturing industry seize the market (3). On the other hand, the effect of pharmaceutical manufacturing originates from the people's inner demand for health. Pharmaceutical manufacturing innovation can maintain and even improve people's health level (4). Glied and Llerasmuney (5) found that many innovations improved people's health from 1970 to 1990, such as improvement of the public water system, the quality of health care, people's cognition of many risk factors of diseases, and so on. This series of technological changes has promoted the improvement of people's health, thus making people live a better life (6). In the global health system, drugs play a significantly important role in diagnosing, treating, and preventing diseases (7). However, pharmaceutical manufacturing has faced the dual challenge of cost and innovation since the mid-1990s. To relieve R&D pressure, more and more pharmaceutical companies are increasingly engaging in open innovation (8). The pharmaceutical industry is a knowledge-intensive industry. The breakthroughs of life science and biotechnology continued to promote the rapid development of the pharmaceutical manufacturing industry (9). Innovations in health science have greatly changed the abilities of treating diseases and improving life quality. In the past few decades, pharmaceutical manufacturing has succeeded in turning science into products (10), which contributed to lengthening people's lives around the world (11). There is no doubt that advances in medical technology can further improve the health of patients, while also reducing the physical risks of healthy consumers who may become ill (12). Therefore, it is significant for us to focus on the relationship between pharmaceutical manufacturing innovation and perceived health. In this paper, pharmaceutical manufacturing innovation refers to drug innovation, including drug innovation in pharmaceuticals and medicinal, chemical, and botanical products.

Pharmaceutical manufacturing innovation affects health in different ways. From the direct influencing factors, the particular features of pharmaceutical manufacturing innovation affect the level of health. Dimasi et al. (13) mentioned that the development of new drugs is a very long and expensive process, which resulted in pharmaceutical manufacturing innovation changing the marginal effect and sensitivity on the level of health. Meanwhile, education as one of the key factors in pharmaceutical manufacturing also affects health. Education and health are considered as the most important components of human capital (14). Their relationship has been paid high attention by economists. Meara et al. (15) found that education makes a huge difference in life expectancy. Moreover, their differences can persist over time if not increased. Among the findings in social science research, there was a strong positive relationship between education and health at all ages (16). In addition, there are differences in health at different ages, which are often determined by the age structure of a country's population. Accordingly, the age structure of the population results in the fact that pharmaceutical manufacturing directly affects the level of health. Nelson and Phelps (17) deemed that the human capital is an important factor determining the technology import and usage of a country. Adequate human capital implies significant technical progress. The human capital shows close relation with the age structure of the population. The relationship between pharmaceutical manufacturing and health also depends on other factors. The existing literature indicates that there is a strong relationship between health and income (18, 19). For example, technical progress usually leads to an increase rather than a reduction in costs (20, 21). It is found that the R&D spending in the pharmaceutical industry has increased significantly, which results in the increase of costs in pharmaceutical manufacturing and health care (22, 23). The cost of health care increases with the continuous promotion of pharmaceutical manufacturing innovation. People have to pay a higher cost when enjoying the welfare brought by pharmaceutical manufacturing. The overall income in a country may partly determine people's enjoyment in the welfare brought by pharmaceutical manufacturing innovation. Hence, the relationship between pharmaceutical manufacturing innovation and health depends on the income.

Even though the impact of pharmaceutical manufacturing innovation on health is an undeniable fact, the impact of pharmaceutical manufacturing on cognitive health has not attracted enough attention. Cognitive health involves not only health conditions relevant to diseases but also some factors like health perception and mental health. Typically, innovative research on vaccines in the pharmaceutical manufacturing industry has an important impact on people's cognitive health assessment, such as the impact of the COVID-19 epidemic. Accordingly, this paper focuses on the effects of pharmaceutical manufacturing innovation on perceived health. The contribution is indicated as follows. First, we study the effect of pharmaceutical manufacturing innovation on perceived health. Most existing literature has suggested the positive effects of pharmaceutical manufacturing innovation on health. However, health is measured by obvious indicators like infant mortality rate. Although these indicators can reflect the real health level, they cannot embody the integration condition involving health level, health cognition, and mental health. Accordingly, we synthetically measure health by the indicator of perceived health and test the effect from the pharmaceutical manufacturing innovation. Second, the heterogeneity effects of pharmaceutical manufacturing innovation on perceived health will be explored. Each core factor affecting the perceived health shows heterogeneity at different levels and degrees. Thus, in this paper, we study heterogeneity based on their classified sample with levels of innovation, aging population, and education. It is found that the effects of pharmaceutical manufacturing innovation on perceived health show significant differences in both degrees and directions with regard to innovation level or degree of aging population and education. Third, we study the influencing mechanism of pharmaceutical manufacturing innovation on perceived health. This is done on the basis of testing the effects of pharmaceutical manufacturing innovation on perceived health. The transmission channel between them is explored. The empirical results suggest that the income level positively moderates the effects of pharmaceutical manufacturing innovation on the perceived health.

The rest of the paper is organized as follows. In section Methods, we introduce the methods used in this paper. Section Data describes the data and variables that we use for this paper. Section Results discusses the empirical results, including heterogeneity analysis of pharmaceutical manufacturing innovation on perceived health and moderating effect between pharmaceutical manufacturing innovation and perceived health. Finally, we conclude the paper in section Conclusions.

## Methods

Pharmaceutical manufacturing innovation affects people's perceived health, which is mainly reflected in two aspects. The first aspect refers to the effect of diseases. Pharmaceutical manufacturing develops new drugs and therapies, which contributes to maintaining people's health (4). Thus, it changes people's perception of their health. The second aspect is the influence of people's expected health. The enhancement of pharmaceutical manufacturing innovation level in a country indeed can strengthen people's confidence in national medical and health services, and then they need not worry about their future health, which results in positive attitudes on their expected perceived health. Pharmaceutical manufacturing innovation contributes not only to treating patients (24) but also to reducing the potential risk brought by diseases to healthy people. Thus, the overall health level is guaranteed.

In this paper, we take both physical and mental health into consideration. We select the perceived health as the indicator to measure health level. This indicator takes the proportion of people older than 15 years who think that they are in good or very good health. The perceived health is a restricted explained variable, and its value ranges from 0 to 1. The linear hypothesis of the model will be broken when the explained variable is restricted, and the estimation of model parameters using the least square method will be biased and inconsistent. In order to avoid this situation, Tobit proposed the censored regression model using the maximum likelihood method in 1958, namely, the Tobit model (25). Therefore, we adopt the panel Tobit model to address the effect taken by pharmaceutical manufacturing innovation on the perceived health in this paper. The panel Tobit model is set as follows:

(1) $$\begin{array}{r} PHE_{i,t-k}=\beta_{0}+\beta_{1}IPM_{it}+\sum \alpha_{i}X_{it}+\varepsilon_{it} \end{array}$$

where the subscripts *i* and *t* represent the country and the year, respectively, and the subscript *k* means that the effect of pharmaceutical manufacturing innovation on perceived health has a *k* years lag. PHE is the explained variable, representing the perceived health. IPM is the explanatory variable, denoting the pharmaceutical manufacturing innovation. *X* represents the control variable, while ε_*it*_ represents the stochastic disturbance.

In addition, as the samples are not enough, it may result in unreliable parameter estimation when estimating parameters of model (1) directly. In order to make full use of the information in the original data and improve the accuracy of estimation, we adopt the Bootstrap method (26) (see Supplementary Material for introduction of bootstrap method) by repeating sampling 500 times with put back in this paper to estimate parameters in model (1). Bootstrap is a feasible and effective method to deal with small sample data (27). Therefore, we adopt the Bootstrap method to repeat sampling before estimating the parameters.

The relationship between pharmaceutical manufacturing innovation and perceived health is also influenced by other factors. Firstly, changes in the age structure of a country will affect the level of technological innovation. As people get older, innovation activity slows down (28, 29). Countries with high aging have relatively low innovation levels, which further affects health. The age structure influences the relationship between pharmaceutical manufacturing innovation and perceived health. Thus, in this paper, we consider the moderating role of age structure between pharmaceutical manufacturing innovation and perceived health. Secondly, different education levels will influence the relationship between technological innovation and perceived health. As a typical R&D industry (1), the pharmaceutical manufacturing industry is in great demand for highly educated talents. In countries with high educational levels, the stock of human capital involved in innovation is relatively sufficient, which promotes innovation and enhances the effect of pharmaceutical manufacturing innovation on perceived health. Hence, in this paper, we also consider the moderating role of educational level between pharmaceutical manufacturing innovation and perceived health. Thirdly, the unemployment rate also affects the relationship between technological innovation and perceived health. According to the results in Vosemer et al. (30), unemployment has a negative effect on health. On the one hand, unemployment reduces the income of individuals or families, which results in a comprehensive influence on health (31). On the other hand, unemployment also causes mental stress, anxiety, and other psychological problems. In countries with high levels of unemployment, it is relatively difficult to improve perceived health through pharmaceutical manufacturing innovation. The effect of pharmaceutical manufacturing innovation on perceived health depends on the unemployment rate. Thus, in this paper, we consider the moderating role of unemployment rate between pharmaceutical manufacturing innovation and perceived health. Many existing works suggest that income is one of the main factors in maintaining a healthy level. Fourthly, income levels may also have an impact on the relationship between technological innovation and perceived health. Carlson (32) pointed that there are many factors affecting health, involving economic factors and social factors, where economic factors seem to predominate. Moreover, Toge (33) proposed that lower income will reduce people's purchasing power for goods, services, and activities, which are beneficial to health, resulting in a negative effect on health. Pharmaceutical manufacturing innovation often leads to an increase in medical costs (23). People with low income may not be able to afford medical expenses. They cannot improve their health by pharmaceutical manufacturing innovation. Thus, in this paper, we consider the moderating role of income level between pharmaceutical manufacturing innovation and perceived health.

From the above, we take age structure, educational level, unemployment rate, and income level as the moderating variables, and analyze their moderating roles in the relationship between pharmaceutical manufacturing innovation and perceived health in this paper. We add interaction terms (34) of age structure, educational level, unemployment rate, and income level into model (1) and then get the following model (2). Before estimating the parameters, we need to centralize explanatory variables and moderating variables and then adopt the Bootstrap method to repeat sampling.

(2) $$\begin{array}{r} PHE_{i,t-k}=\beta_{0}+\beta_{1}IPM_{it}+\beta_{2}Moderate_{it}+\lambda_{i}IPM_{it}\\ \;\times \;Moderate_{it}+\sum \alpha_{i}X_{it}+\varepsilon_{it} \end{array}$$

where subscripts *i* and *t* represent the country and the year and subscript *k* represents the time lag of effect taken by pharmaceutical manufacturing innovation on perceived health, which is set to be *k* = 4. PHE is the explained variable, representing perceived health. IPM is the explanatory variable, representing the pharmaceutical manufacturing innovation. Moderate is the moderating variable; IPM × Moderate is the interaction term representing pharmaceutical manufacturing innovation crossing the moderating variable. *X* is the control variable, while ε_*it*_ is the stochastic disturbance.

## Data

To ensure data integrity, this paper takes 22 OECD countries including Austria, Belgium, Canada, Czech Republic, Denmark, Estonia, Finland, France, Germany, Hungary, Israel, Italy, Lithuania, Netherlands, Norway, Poland, Portugal, Slovak Republic, Spain, Sweden, Turkey, and United States as its research targets from 2010 to 2017. The variables, methodologies, and data sources are shown in Table 1. Perceived health is the explained variable, measured by the proportion of people who are beyond 15 years old and who think that they are in good health or very good health. The pharmaceutical manufacturing innovation is the explanatory variable, measured by R&D expenditures of pharmaceuticals and medicinal, chemical, and botanical products. There are many factors affecting health. When setting an econometric model, we have to assume that all other factors affecting health are constant; namely, other influencing factors are controlled in quantitative research, and these influencing factors are set as control variables. In this paper, the control variables are selected according to relevant theories and empirical results (16, 33, 35–39). By summarizing the existing empirical results, together with features of considered objects, four control variables are selected in this paper, including age structure, educational level, unemployment rate, and income level. Due to the missing data on educational level from some countries in some specific years, we interpolate the missing data by adopting linear regression model on the basis of the features of the original data. In addition, we select the income level as the moderate variable to explore the influencing mechanism between the pharmaceutical manufacturing innovation and the perceived health.

**Table 1:** Data and variables.

**Nature of variables**	**Variable**	**Abbreviation**	**Measurement**	**Source**
Dependent variables	Perceived health	PHE	Good/very good health, total aged 15+ (% of population)	OECD statistics
Explanatory variable	Innovation level of pharmaceutical manufacturing	IPM	R&D expenditures of Pharmaceuticals, medicinal, chemical, and botanical products (constant 2015 US$)	OECD statistics
Control variables and moderating variables	Age structure	AST	65 years old and over (% of total population)	OECD Statistics
	Level of education	LED	Mean years of schooling, population 25+ years	The UNESCO Institute for Statistics
	Unemployment	UEM	Unemployment, total (% of total labor force)	World Bank
	GNI per capita	GNIPC	GNI per capita (constant 2010 US$)	World Bank

## Results

### Descriptive Statistics and Pairwise Correlations

Table 2 reports descriptive statistics. Overall, the minimum of PHE is 42.6, while the maximum is 89. The mean is 68.2068 and the median is 68.65 (omitted in Table 2 because of space constraint). This suggests that PHE is almost not skewed. The minimum of IPM is 0.0002, while the maximum is 66.2020. The mean is 6.1880, which suggests a lower level with greater differences among samples. In addition, we present descriptive statistics for sub-samples divided by pharmaceutical manufacturing innovation level, age structure, and educational level. The dividing criterion for samples is as follows. Calculate and rank the annual average of R&D expenditure of pharmaceutical manufacturing industry in each country and then divide the entire sample into two groups of sub-samples by the median for a high innovation level and a low innovation level. Calculate and rank the annual average of age structure in each country and then divide the entire sample into two groups of sub-samples by the median for high aging population and low aging population. Calculate and rank the annual average of education years in each country and then divide the entire sample into two groups of sub-samples by the median for high educational level and low educational level.

**Table 2:** Descriptive statistics.

	**Variable**	**Obs**	**Mean**	**Std. dev**.	**Min**	**Max**
Full sample	PHE	176	68.2068	11.4209	42.6000	89.0000
	IPM	176	6.1880	15.4455	0.0002	66.2020
	AST	176	16.7955	3.2119	7.0000	22.3000
	LED	176	12.0413	1.6031	7.2581	14.2804
	UEM	176	8.7137	4.1186	2.8900	26.0940
	GNIPC	176	3.7050	1.9760	1.0582	9.5345
H_IPM	PHE	88	71.3250	8.8116	55.0000	88.1000
	IPM	88	12.1778	20.1806	0.5116	66.2020
	AST	88	17.4546	2.8955	9.9000	22.3000
	LED	88	12.0834	1.2734	9.4173	14.2804
	UEM	88	8.8206	4.7463	2.8900	26.0940
	GNIPC	88	3.9289	1.4294	1.2519	6.4128
L_IPM	PHE	88	65.0886	12.8476	42.6000	89.0000
	IPM	88	0.1983	0.1753	0.0002	0.7205
	AST	88	16.1364	3.3894	7.0000	21.1000
	LED	88	11.9992	1.8827	7.2581	13.9619
	UEM	88	8.6068	3.4015	3.1230	17.8140
	GNIPC	88	3.4812	2.3895	1.0582	9.5345
H_AST	PHE	88	64.2318	10.5725	42.6000	79.8000
	IPM	88	2.0299	3.0166	0.0002	10.5123
	AST	88	18.9398	1.3673	16.3000	22.3000
	LED	88	11.8601	1.6649	7.9570	14.2804
	UEM	88	9.9376	4.8939	3.7460	26.0940
	GNIPC	88	3.7906	1.5211	1.1876	6.4128
L_AST	PHE	88	72.1818	10.8918	55.0000	89.0000
	IPM	88	10.3462	20.8756	0.0021	66.2020
	AST	88	14.6511	3.0950	7.0000	18.8000
	LED	88	12.2226	1.5268	7.2581	13.8533
	UEM	88	7.4898	2.6721	2.8900	14.3790
	GNIPC	88	3.6195	2.3507	1.0582	9.5345
H_LED	PHE	88	68.7136	13.7088	42.6000	89.0000
	IPM	88	6.5024	15.8501	0.0002	66.2020
	AST	88	16.5784	2.8017	9.9000	21.2000
	LED	88	13.1299	0.5317	12.1523	14.2804
	UEM	88	7.1952	2.7803	2.8900	17.8140
	GNIPC	88	4.0064	2.3148	1.1876	9.5345
L_LED	PHE	88	67.7000	8.5979	45.9000	79.8000
	IPM	88	5.8737	15.1143	0.0021	56.0604
	AST	88	17.0125	3.5785	7.0000	22.3000
	LED	88	10.9528	1.5779	7.2581	12.9329
	UEM	88	10.2322	4.6611	4.1560	26.0940
	GNIPC	88	3.4037	1.5205	1.0582	5.8229

*H_IPM and L_IPM represent, respectively, the countries with high and low pharmaceutical manufacturing innovation levels; H_AST and L_AST represent, respectively, the countries with high and low aging population; H_LED and L_LED represent, respectively, the countries with high and low educational levels*.

Table 3 shows the Pearson correlation matrix, and the results report the correlation between explanatory variables and control variables in the model with PHE. We can find that pharmaceutical manufacturing innovation is positively related to perceived health in the full sample. However, the relationship between pharmaceutical manufacturing innovation and perceived health is different in different sub-samples.

**Table 3:** Pearson correlation matrix.

	**Variable**	**IPM**	**AST**	**LED**	**UEM**	**GNIPC**
Full sample	PHE	0.1572^**^	−0.2924^***^	0.1877^**^	−0.2510^***^	0.6497^***^
H_IPM		0.0916	−0.4943^***^	0.1718	0.0072	0.5722^***^
L_IPM		0.7635^***^	−0.3013^***^	0.1951^*^	−0.5657^***^	0.6800^***^
H_AST		0.5079^***^	−0.0481	0.0075	−0.0774	0.8210^***^
L_AST		0.0271	−0.1079	0.3202^***^	−0.3401^***^	0.6632^***^
H_LED		0.4571^***^	−0.5209^***^	0.0809	−0.4298^***^	0.6394^***^
L_LED		−0.3376^***^	−0.0438	0.3839^***^	−0.1449	0.6804^***^

*H_IPM and L_IPM represent, respectively, the countries with high and low pharmaceutical manufacturing innovation levels; H_AST and L_AST represent, respectively, the countries with high and low aging population; H_LED and L_LED represent, respectively, the countries with high and low educational levels*.

*** *p < 0.01*,

** *p < 0.05, and*

* *p < 0.1*.

From the sub-samples of different levels of innovation in pharmaceutical manufacturing in Table 2, the mean of PHE of H_IPM is 71.3250, higher than that of L_IPM, which is given as 65.0886. From the overall level of sub-samples, the innovation level of pharmaceutical manufacturing industry is positively correlated with the perceived health level. Combined with Table 3, IPM is positively related to PHE in L_IPM, but the cross-correlation between IPM and PHE in H_IPM is not significant. Accordingly, the effect of pharmaceutical manufacturing innovation on perceived health shows significant difference. From the age structure, the mean of PHE of H_AST is 64.2318, lower than that of L_AST, which is given as 72.1818. The mean of IPM of H_AST is 2.0299, lower than that of L_AST, which is given as 10.3462. In Table 3, IPM is positively related to PHE in H_AST, and the cross-correlation between IPM and PHE in L_AST is positive but not significant. Accordingly, the effect of pharmaceutical manufacturing innovation on perceived health may have significant differences in the sub-samples divided by age structure. From the educational level, the mean of PHE of H_LED is 68.7136, close to that of L_LED, given as 67.7000. However, the mean of IPM of H_LED is 6.5024, higher than that of L_LED, given as 5.8737. As shown in Table 3, IPM is positively related to PHE in H_LED but negatively related to PHE in L_LED. Accordingly, the higher educational level implies the higher levels of both the pharmaceutical manufacturing innovation and perceived health. The effect between them may be different in countries with different educational levels.

We aim to explore the effect of pharmaceutical manufacturing innovation on perceived health. The unit root test is performed to check whether the variables are stationary before their effect exploration. Table 4 reports the results for a battery of panel unit root tests for PHE, IPM, AST, LED, UEM, and GNIPC. Two common methods are used to test data stationarity in this paper. The first is the Levin-Lin-Chu unit-root test (40); the second is the standard Augmented Dickey–Fuller *t*-test (41). In particular, we report results from two tests of the null hypothesis that each series contains a unit root. In the two cases, we find a uniform conclusion that the null hypothesis of non-stationarity can be strongly rejected at a 1% significance level. Consequently, we can conclude that all variables are stationary.

**Table 4:** Results of panel unit root test.

	**LLC**	**Fisher-ADF**
PHE	−14.8200^***^	103.7624^***^
IPM	−12.1826^***^	71.3256^***^
AST	−7.2738^***^	88.8233^***^
LED	−22.9360^***^	93.1054^***^
UEM	−13.8784^***^	110.3657^***^
GNIPC	−8.4407^***^	117.4376^***^

*LLC denotes Levin-Lin-Chu unit-root test; Fisher-ADF denotes fisher ADF unit-root tests*;

*** *p < 0.01*.

Lag effect should be taken into consideration when testing the impact of pharmaceutical manufacturing innovation on perceived health. There should be a lag effect between pharmaceutical manufacturing innovation and perceived health, because there is also a time lag from the moment when a drug was approved to the moment when it really worked (42, 43). Lichtenberg (44) found that premature mortality in Canada is associated with at least 10-year cumulative use of drugs. Lichtenberg (45) presented a negative relationship between cancer mortality and the more than 5-year cumulative drugs by estimating 19 cancers from 36 countries and the quantity of drugs responding to these cancers. Thus, the effect of pharmaceutical manufacturing innovation on perceived health may have several years' delay, which cannot be clearly determined. In this paper, we make a tentative attempt and test on the lag effect of 1–4 years, respectively, between innovation in pharmaceutical manufacturing and perceived health. Furthermore, the effect of pharmaceutical manufacturing innovation on perceived health is empirically analyzed by using the panel Tobit model. Parameter estimation results can be seen in Table 5.

**Table 5:** The effect of pharmaceutical manufacturing innovation on perceived health.

**Dependent variable = PHE**	**Tobit A**	**Tobit B**	**Tobit C**	**Tobit D**
IPM	0.0600^*^	0.0649^**^	0.0789^***^	0.0775^***^
	(0.0081)	(0.0098)	(0.0079)	(0.0057)
AST	−0.0531	−0.3044	−0.5972^***^	−1.1090^***^
	(0.0289)	(0.0317)	(0.0299)	(0.0261)
LED	−0.2329	−0.0538	0.4916	1.0046^**^
	(0.0546)	(0.0565)	(0.0600)	(0.0506)
UEM	0.2160^**^	0.2162^**^	0.1625	0.1838
	(0.0073)	(0.0092)	(0.0134)	(0.0182)
GNIPC	3.4145^***^	4.1546^***^	4.1236^***^	3.9083^***^
	(0.0730)	(0.0459)	(0.0398)	(0.0383)
Constant	56.9077^***^	56.1598^***^	55.0621^***^	58.2989^***^
	(0.5507)	(0.6289)	(0.8872)	(0.8327)
sigma_u	1.4080^***^	1.2832^***^	1.1754^***^	1.0626^***^
	(0.1560)	(0.1221)	(0.0945)	(0.0771)
sigma_e	0.0798^***^	0.0807^***^	0.0937^***^	0.1133^***^
	(0.0064)	(0.0065)	(0.0080)	(0.0102)
Observations	154	132	110	88

*The explained variables in model Tobit A–D are the Perceived health with 1-, 2-, 3-, and 4-year delay, respectively. Repeating the samples 500 times by the Bootstrap method is carried out before taking regression. Standard errors in parentheses*;

*** *p < 0.01*,

** *p < 0.05*,

* *p < 0.1*.

The parameter estimation results in Table 5 indicate that there is a positive effect between pharmaceutical manufacturing innovation and perceived health. In addition, the pharmaceutical manufacturing innovation 4 years ago makes the largest differences to the perceived health. The coefficients of pharmaceutical manufacturing innovation are all positive and remain almost the same regardless of the lag years, which suggests a robust estimation. When the lag years between pharmaceutical manufacturing innovation and perceived health are 1, 2, 3, and 4 in order, the coefficients of pharmaceutical manufacturing innovation are 0.0600, 0.0649, 0.0789, and 0.0775 correspondingly, which are significantly positive at the 10% level. It suggests that the promotion of pharmaceutical manufacturing innovation can enhance the perceived health. From the regression results in Table 5, the estimation is the best when the lag between pharmaceutical manufacturing innovation and perceived health is set to be 4 years (in the regression of panel Tobit model in sections Data and Results, the perceived health with a 4-year delay is set as the explained variable). Besides, with regard to the regression results of the panel Tobit model where the explained variable is the perceived health with a 4-year delay, the coefficient of age structure is significantly negative at the 10% level, which suggests the negative effect of the age structure on perceived health. The coefficients of both educational level and income are significantly positive at the 10% level, which suggests that the increase of educational level and income will enhance the perceived health level.

### Heterogeneity in Effect of Pharmaceutical Manufacturing Innovation on Perceived Health

Although the above results show the positive effect of pharmaceutical manufacturing innovation on perceived health, this effect may be different in different conditions. In this section, we examine the heterogeneity for the effect of pharmaceutical manufacturing innovation on perceived health with the entire sample divided into different conditions (46).

The effect of pharmaceutical manufacturing innovation on perceived health may be different in countries with different pharmaceutical manufacturing innovation levels, aging degrees of population, or educational levels. First, as an industry based on R&D, the pharmaceutical manufacturing industry can win the market competition by paying attention to technological innovation, as well as owning high-quality and cutting-edge creations (47, 48). Although R&D innovation is important to the pharmaceutical manufacturing industry, different countries have different R&D intensities, which results in countries with different pharmaceutical manufacturing innovation levels showing different effects on health. From Table 2, higher pharmaceutical manufacturing innovation leads to higher perceived health. It is relatively difficult to improve health by pharmaceutical manufacturing innovation. However, for countries with low pharmaceutical manufacturing innovation, there is plenty of space for improving health through pharmaceutical manufacturing innovation. Accordingly, the sample is divided into two sub-samples: one represents high-innovation countries and the other represents low-innovation countries. Second, some existing results have shown that the aging of population is relevant to technical innovation. Clearly, countries with high levels of aging have a higher proportion of elderly people than countries with low levels of aging. There are more health problems among the elderly. In addition, Meyer (49) found that the establishment of high-tech companies is highly correlated with the age structure of the region in which they are located. From Table 2, due to the age structure, in countries with high levels of aging, the pharmaceutical manufacturing innovation and perceived health are both significantly lower than countries with low levels of aging. Thus, countries with different levels of aging show different effects of pharmaceutical manufacturing innovation on perceived health. Accordingly, the sample is divided into two sub-samples: one represents countries with high levels of aging and the other represents countries with low levels of aging. Third, education can significantly improve an individual's health status, which is considered as an important factor affecting individual health (50, 51). Smith (52) found that the higher the educational level, the higher the level of individual health. From Table 2, in high-educational-level countries, the pharmaceutical manufacturing innovation and perceived health are both higher than those in countries with low educational levels. People in countries with high educational levels are more willing to accept changes brought by pharmaceutical manufacturing innovation and enjoy more the advantages of such changes. Thus, the effect of pharmaceutical manufacturing innovation on the perceived health may be heterogeneous in the countries with different educational levels. Accordingly, the sample is divided into two sub-samples: one represents the countries with high educational levels and the other represents the countries with low educational levels.

On the basis of theoretical analysis, the overall sample in this paper is divided according to pharmaceutical manufacturing innovation level, aging of population, and educational level. Parameter estimation results are shown in Table 6 by the Bootstrap method repeating sampling and panel Tobit regression.

**Table 6:** Heterogeneity in effect of pharmaceutical manufacturing innovation on perceived health.

**Dependent variable = PHE**	**Innovation level**		**Aging level**		**Educational level**	
	**High level**	**Low level**	**High level**	**Low level**	**High level**	**Low level**
IPM	0.0280	15.6326^**^	−0.4293^*^	0.0267	0.1641^***^	−0.0367
	(0.0082)	(1.4551)	(0.0692)	(0.0080)	(0.0132)	(0.0124)
AST	−1.1191^***^	−1.4356^***^	−0.8952	−1.4417^***^	−1.9193^***^	−0.9301^***^
	(0.0667)	(0.0606)	(0.1013)	(0.0827)	(0.1402)	(0.0609)
LED	0.9215	1.2645^***^	0.1969	3.0625^***^	3.0291	1.4420^***^
	(0.1976)	(0.1227)	(0.1303)	(0.1786)	(0.6038)	(0.1326)
UEM	0.1666	0.2120	0.6520^*^	−0.2294	0.1933	0.2414
	(0.0418)	(0.0346)	(0.0540)	(0.0413)	(0.0630)	(0.0211)
GNIPC	4.3199^***^	3.0442^***^	7.0121^***^	2.5263^***^	3.3799^***^	4.6294^***^
	(0.1500)	(0.0710)	(0.1420)	(0.0813)	(0.1048)	(0.1698)
Constant	60.9693^***^	57.9708^***^	47.1624^**^	47.8398^***^	45.2931	49.5699^***^
	(3.5204)	(1.2344)	(3.2319)	(2.2456)	(7.5280)	(1.4762)
sigma_u	1.2654^***^	1.3904^***^	0.9517^***^	1.5638^***^	1.5424^***^	1.0221^***^
	(0.2349)	(0.2304)	(0.2018)	(0.2025)	(0.4069)	(0.1840)
sigma_e	0.1321^***^	0.1871^***^	0.1595^***^	0.1456^***^	0.1769^***^	0.1495^***^
	(0.0168)	(0.0229)	(0.0246)	(0.0195)	(0.0262)	(0.0172)
Observations	44	44	44	44	44	44

*The explained variable is the Perceived health with 4-year delay. Standard errors in parentheses*;

*** *p < 0.01*,

** *p < 0.05, and*

* *p < 0.1*.

Table 6 reports the specific effect of pharmaceutical manufacturing innovation on perceived health from each sub-sample. In the regression groups of high and low innovation levels, the effect of pharmaceutical manufacturing innovation on perceived health shows heterogeneity. The regression coefficients of pharmaceutical manufacturing innovation are 0.0280 and 15.6326, respectively. The former does not pass the significant test while the latter passes the 5% significance test. Thus, in countries with low pharmaceutical manufacturing innovation, pharmaceutical manufacturing innovation can promote perceived health. Compared with the results in the overall sample, the effect of pharmaceutical manufacturing innovation on perceived health in countries with low innovation levels has been greatly improved. This may be due to the fact that with the improvement of pharmaceutical manufacturing innovation, the marginal effect on health will decrease. Therefore, the effect is greater in countries with low innovation level.

In the regression groups of high and low aging, the effect of pharmaceutical manufacturing innovation on perceived health also shows heterogeneity. The regression coefficients of pharmaceutical manufacturing innovation are −0.4293 and 0.0267. The former passes the 10% significance test, while the latter does not pass the test. Thus, in countries with high levels of aging, pharmaceutical manufacturing innovation shows negative effect on perceived health. There may be two reasons. On the one hand, the demographic change of labor force is closely relevant to innovation capacity of the country (53, 54). To be specific, there is a hump relation between age and technological innovation. The peak of technological innovation belongs to the age range from 35 to 40 years old. With increasing age, innovation activity slows down (28, 29). In countries with high levels of aging, the stock of young human capital is relatively small, which lowers the efficiency of pharmaceutical manufacturing innovation. It may further weaken the effect of innovation on health. On the other hand, the health of the elderly is relatively poor. There are great difficulties in improving health in countries with high levels of aging. Hence, in countries with high levels of aging, when the decline magnitude in perceived health associated with age exceeds the positive effect of pharmaceutical manufacturing innovation on perceived health, pharmaceutical manufacturing innovation can actually reduce the perceived health.

In the regression groups of high and low educational levels, the effect of pharmaceutical manufacturing innovation on perceived health also reflects heterogeneity. The regression coefficients of pharmaceutical manufacturing innovation are 0.1641 and −0.0367. The former passes the 1% significance test, while the latter does not pass the test. Thus, in countries with high educational levels, pharmaceutical manufacturing innovation contributes to improving perceived health. Compared with the results in the overall sample, the effect from pharmaceutical manufacturing innovation was increased by 111.74%. It may be because people with high educational levels are more able to make use of the information in health care and medicine. Meanwhile, they can be a better fit for complicated medical treatments and benefit more from pharmaceutical manufacturing innovation (55).

### Moderating Effect Between Pharmaceutical Manufacturing Innovation and Perceived Health

The pharmaceutical manufacturing innovation shows positive effects on perceived health. The empirical results in this section further answer the question “Why does the pharmaceutical manufacturing innovation show positive effect on perceived health?” Parameter estimation results are shown in Table 7.

**Table 7:** Moderating effect between pharmaceutical manufacturing innovation and perceived health.

**Dependent variable = PHE**	**(1)**	**(2)**	**(3)**	**(4)**
IPM	0.0716^***^	−0.0061	0.0259	0.0596^**^
	(0.0049)	(0.0071)	(0.0096)	(0.0044)
AST	−1.0838^***^	−1.1331^***^	−1.1662^***^	−1.1043^***^
	(0.0280)	(0.0247)	(0.0254)	(0.0246)
LED	1.0551^**^	1.4377^***^	1.1196^***^	0.9314^**^
	(0.0524)	(0.0502)	(0.0525)	(0.0495)
UEM	0.1957	0.1970	0.1659	0.1837
	(0.0193)	(0.0183)	(0.0187)	(0.0179)
GNIPC	3.9284^***^	3.7572^***^	3.9007^***^	3.8956^***^
	(0.0377)	(0.0393)	(0.0398)	(0.0389)
IPM × AST	0.0104			
	(0.0040)			
IPM × LED		0.0796^*^		
		(0.0106)		
IPM × UEM			−0.0108^*^	
			(0.0024)	
IPM × GNIPC				0.0415^***^
				(0.0031)
Constant	39.3799^***^	71.4591^***^	59.8499^***^	74.0685^***^
	(0.6756)	(0.4879)	(0.7661)	(0.7934)
sigma_u	1.0818^***^	1.0460^***^	1.0587^***^	1.0387^***^
	(0.0816)	(0.0849)	(0.0814)	(0.0832)
sigma_e	0.1132^***^	0.1133^***^	0.1121^***^	0.1134^***^
	(0.0103)	(0.0101)	(0.0102)	(0.0101)
Observations	88	88	88	88

*The explained variable is the Perceived health with 4-year delay. Standard errors in parentheses*;

*** *p < 0.01*,

** *p < 0.05*,

* *p < 0.1*.

The parameter estimation results in Table 7 indicate that the income can moderate the relationship between pharmaceutical manufacturing innovation and perceived health. As shown in Table 7, the coefficients of interaction terms between pharmaceutical manufacturing innovation and age structure, between pharmaceutical manufacturing innovation and educational level, and between pharmaceutical manufacturing innovation and unemployment rate are not significant, which suggests that the age structure, educational level, and unemployment rate do not play moderating roles in the relationship between pharmaceutical manufacturing innovation and perceived health. The coefficient of interaction term between pharmaceutical manufacturing innovation and income is 0.0415, which is significant at the 1% level, suggesting that the income can positively moderate the relationship between pharmaceutical manufacturing innovation and perceived health. On the one hand, pharmaceutical manufacturing innovation is a long and difficult process. Generally, there is only 1 in 10,000 products that can be put into the market (7). In addition, according to the results of Dimasi et al. (56), the annual growth rate of drug development cost is 7.4%, which is higher than the inflation rate. Thus, pharmaceutical manufacturing innovation has led to a series of increases in medical costs. On the other hand, pharmaceutical manufacturing innovation not only improves health but also increases financial risk. Once people get sick, their burden of paying expensive medical bills will increase (12). For high-income people, they can afford the costs of pharmaceutical manufacturing innovation. They benefit more from pharmaceutical manufacturing innovation and improve perceived health. For low-income people, they can only benefit finitely from pharmaceutical manufacturing innovation because of their financial ability, which results in low improvement of the perceived health.

## Conclusions

In view of selecting 22 OECD countries from 2010 to 2017 as samples, we study the effect of pharmaceutical manufacturing innovation on perceived health by using the Tobit model in this paper. At the same time, this paper considers heterogeneity effects of pharmaceutical manufacturing innovation on perceived health in countries with different pharmaceutical manufacturing innovation levels, aging of population, and educational levels. In addition, the moderating effect of income is verified by using the Tobit model with moderating effect. Some conclusions are as follows:

First, pharmaceutical manufacturing innovation 4 years ago has a positive effect on perceived health. People's perceived health improves with the improvement of pharmaceutical manufacturing innovation. In this paper, we verify the positive effect of pharmaceutical manufacturing innovation 4 years ago on physical health and expected health, which may be ascribed to two origins. On the one hand, pharmaceutical manufacturing innovation has brought new and efficient medical methods, which are beneficial to the diagnosis, prevention, and treatment of diseases and thus improve people's health. On the other, pharmaceutical manufacturing innovation also means the progress of medical and health services, which boosts people's confidence in the country's medical standards and thus improves people's expected health.

Second, the heterogeneous effects of pharmaceutical manufacturing innovation on perceived health are mainly reflected in the innovation level, aging degree, and educational level, as well as even negative effects in countries with high levels of aging. Pharmaceutical manufacturing innovation in countries with low innovation levels shows positive effects on perceived health, while there is no evidence suggesting the relationship between pharmaceutical manufacturing innovation and perceived health in countries with high innovation levels. The pharmaceutical manufacturing innovation in countries with high aging shows a negative effect on perceived health, while there is no evidence suggesting the relationship between pharmaceutical manufacturing innovation and perceived health in countries with low aging. The pharmaceutical manufacturing innovation in countries with high incomes shows a positive effect on perceived health, while there is no evidence suggesting the relationship between pharmaceutical manufacturing innovation and perceived health in countries with low incomes.

Third, the income can moderate the effect of pharmaceutical manufacturing innovation on perceived health. On the one hand, pharmaceutical manufacturing innovation usually goes through a long process. In addition, the introduction of new drugs also requires rigorous scrutiny, and their percentage of drug usage is relatively low, which leads to a continuously increasing cost for pharmaceutical manufacturing innovation. On the other hand, pharmaceutical manufacturing innovation improves health care, but at the same time, it also increases treatment cost. For high-income people, they are able to afford the expensive treatment costs and can get benefits from pharmaceutical manufacturing innovation.

Our findings present important practical significance for government and managers. First, technological development is the main driving force for innovation (57). We can enhance the intensity of R&D and drive pharmaceutical manufacturing innovation. Especially for countries with low pharmaceutical manufacturing innovation and high educational levels, national health can be improved by accelerating pharmaceutical manufacturing innovation. Second, we should take the increasing treatment cost into consideration while encouraging pharmaceutical manufacturing innovation. Medical subsidies can be actively adopted, especially for people with lower incomes, and then more people can benefit from pharmaceutical manufacturing innovation. Third, pharmaceutical manufacturing innovation can not only improve national health but also increase people's confidence on the national medical and health system, especially when the macro environment changes (58). When we are hit hard by major public health events, such as the COVID-19 outbreak, a strong health system can undoubtedly increase people's trust in the country and greatly reduce social panic. Thus, the nation is more able to respond to the impact of public health emergencies.

This study takes lag effect into consideration and verifies the positive effect of pharmaceutical manufacturing innovation 4 years ago on perceived health. At the same time, we also find that the heterogeneous effects of pharmaceutical manufacturing innovation on perceived health are mainly reflected in the innovation level, aging degree, and educational level. In addition, the income plays a moderate role in the effect of pharmaceutical manufacturing innovation on perceived health. However, this study is not without limitations. To ensure data integrity, this study considers 22 OECD countries as its research targets from 2010 to 2017. This paper adopts the Bootstrap method to improve the accuracy of estimation because the samples are not enough. However, the results would be more appropriate if the sample size was adequate. Besides, the research objects of this study mostly are developed countries that tend to reflect the relationship between pharmaceutical manufacturing innovation and perceived health in developed countries. Therefore, the findings of this study cannot well reflect the effect of pharmaceutical manufacturing innovation on perceived health in other countries.

## Data Availability Statement

The raw data supporting the conclusions of this article will be made available by the authors, without undue reservation.

## Author Contributions

ZL and XL conceived and designed the study and supervised the study. ZL provided guidance and approved the final manuscript. Both authors proposed and discussed the idea of the study and contributed to manuscript writing and editing.

## Conflict of Interest

The authors declare that the research was conducted in the absence of any commercial or financial relationships that could be construed as a potential conflict of interest.
