# Supplementary material for: Will Innovation of Pharmaceutical Manufacturing Improve Perceived Health?
Source: Front Public Health. 2021 Apr 29;9:647357. doi: 10.3389/fpubh.2021.647357 (PMC8116498; doi:10.3389/fpubh.2021.647357)
Supplement: Supplementary file 1 [file Data_Sheet_1.docx]

Supplementary Material

Bootstrap method was proposed systematically for the first time by Efron (1979). Bootstrap method is any test or metric that uses random sampling with replacement, and falls under the broader class of resampling methods. The basic idea of bootstrap method is resampling with replacement from the sample. It replicates observed information according to the original observations, and it is unnecessary to make distribution hypothesis or add information of new sample. But it can make statistical inference on the distribution characteristics of the population by using information from resampling. This technique allows estimation of the sampling distribution of almost any statistic using random sampling methods.

To illustrate bootstrap method, the specific inference process of bootstrap method is as follows:

Firstly, suppose that we have a dataset containing N observations and an estimator that, when applied to the data, produces certain statistics.

Secondly, we draw, with replacement, N observations from the N-observation dataset. In this random drawing, some of the original observations will appear once, some more than once, and some not at all.

Finally, using the resampled dataset, we apply the estimator and collect the statistics. This process is repeated many times; each time, a new random sample is drawn and the statistics are recalculated.

More formally, the bootstrap works by treating inference of the true probability distribution $\boldsymbol{F}$, given the original data, as being analogous to inference of the empirical distribution $\hat{\boldsymbol{F}}$, given the resampled data. The accuracy of inferences regarding $\hat{\boldsymbol{F}}$ using the resampled data can be assessed because we know $\hat{\boldsymbol{F}}$. If $\hat{\boldsymbol{F}}$ is a reasonable approximation to $\boldsymbol{F}$, then the quality of inference on $\boldsymbol{F}$ can in turn be inferred. It is unnecessary that make a hypothesis about population distribution or derive the analytic expression of the estimator in advance for bootstrap method. Reconstructing the sample and continuously calculating the estimated value is the important work that it needs to do. Obviously, it is a non-parametric method in nature.

Multiple resampling builds a dataset of replicated statistics. From these data, we can calculate the standard error by using the standard formula for the sample standard deviation. The formula of standard error is as follow:

$$\hat{\boldsymbol{se}}\boldsymbol{=}\left\{ \frac{\boldsymbol{1}}{\boldsymbol{k-1}}\sum\left( {\hat{\boldsymbol{\theta}}}_{\boldsymbol{i}}\boldsymbol{-}\bar{\boldsymbol{\theta}} \right)^{\boldsymbol{2}} \right\}^{\boldsymbol{2}}$$

where $\hat{\theta}_{i}$ is the statistic calculated using the ith bootstrap sample and k is the number of replications. Although the average, $\bar{\theta}$, of the bootstrapped estimates is used in calculating the standard deviation, it is not used as the estimated value of the statistic itself. Instead, the original observed value of the statistic, $\hat{\theta}_{i}$, is used, meaning the value of the statistic computed using the original N observations.
